# Central 5-HTergic hyperactivity induces myalgic encephalomyelitis/chronic fatigue syndrome (ME/CFS)-like pathophysiology

**DOI:** 10.1186/s12967-023-04808-x

**Published:** 2024-01-08

**Authors:** Jin-Seok Lee, Ji-Yun Kang, Samuel-Young Park, Seung-Ju Hwang, Sung-Jin Bae, Chang-Gue Son

**Affiliations:** 1 Research Center for CFS/ME, Daejeon Hospital of Daejeon University, Daejeon, Republic of Korea; 2 Institute of Bioscience & Integrative Medicine, Daejeon University, Daejeon, Republic of Korea; 3 Korean Medical College of Daejeon University, Daejeon, Republic of Korea; 4 College of Medicine, Kosin University, Busan, Republic of Korea

**Keywords:** Myalgic encephalomyelitis/chronic fatigue syndrome, Fatigue, serotonin, 5-HT_1A_ receptor, Desensitization, Hypocortisolism

## Abstract

**Objectives:**

Myalgic encephalomyelitis/chronic fatigue syndrome (ME/CFS) is a significant medical challenge, with no indisputable pathophysiological mechanism identified to date.

**Methods:**

Based on clinical clues, we hypothesized that 5-hydroxytryptamine (5-HT) hyperactivation is implicated in the pathogenic causes of ME/CFS and the associated symptoms. We experimentally evaluated this hypothesis in a series of mouse models.

**Results:**

High-dose selective serotonin reuptake inhibitor (SSRI) treatment induced intra- and extracellular serotonin spillover in the dorsal raphe nuclei of mice. This condition resulted in severe fatigue (rota-rod, fatigue rotating wheel and home-cage activity tests) and ME/CFS-associated symptoms (nest building, plantar and open field test), along with dysfunction in the hypothalamic-pituitary-adrenal (HPA) axis response to exercise challenge. These ME/CFS-like features induced by excess serotonin were additionally verified using both a 5-HT synthesis inhibitor and viral vector for Htr1a (5-HT_1A_ receptor) gene knockdown.

**Conclusions:**

Our findings support the involvement of 5-HTergic hyperactivity in the pathophysiology of ME/CFS. This ME/CFS-mimicking animal model would be useful for understanding ME/CFS biology and its therapeutic approaches.

**Supplementary Information:**

The online version contains supplementary material available at 10.1186/s12967-023-04808-x.

## Introduction

Myalgic encephalomyelitis/chronic fatigue syndrome (ME/CFS) represents the most debilitating form of medically unexplained chronic fatigue [1, 2]. This disorder affects individuals across all ages, racial, ethnic, and socioeconomic groups with an approximate prevalence rate of 0.9% [3]. ME/CFS is associated with core symptoms, including unrefreshing sleep, post-exertional malaise (PEM), and cognitive impairment, significantly compromising the health-related quality of life in patients [1]. For instance, 25 to 29% of patients experience a house- or bed-bound state, and approximately 21% are unemployed [4]. Moreover, individuals with ME/CFS face a 7-fold higher risk of suicide compared to healthy subjects [5]. However, despite these challenges, no approved effective treatment currently exists [6].

The Institute of Medicine (IOM) has defined this disorder as a complex multisystem neurological disorder; however, the etiology and pathophysiology of ME/CFS remain unclear. Numerous scientists have investigated viral infection, autoimmune dysregulation, abnormal neuroendocrine or neuroinflammation [7]. Recently, novel findings such as an elevated level of transforming growth factor (TGF)-β [8], an altered composition of the gut microbiome [9], and impairment of mitochondria [10] have been reported. Nevertheless, these studies have not fully elucidated the clinical complexity of ME/CFS in patients. On the other hand, some clinical observations have given rise to the proposal of the ‘hyper-serotonergic hypothesis’ as a putative pathophysiology of ME/CFS. This hypothesis is characterized by a low binding potential for the 5-hydroxytryptamine (5-HT)_1A_ receptor or 5-HT transporter on positron emission tomography (PET) analysis [11, 12].

Central serotonin hyperactivity could elucidate the complex clinical features observed in ME/CFS patients, such as sustained fatigue, PEM, hyperalgesia, and poor sleep quality [13, 14]. The 5-HT projection into the hypothalamus play a crucial role in the regulation of glucocorticoid response system [15]. Glucocorticoids generally help organisms in coping with stress and environmental changes, as well as in regulating the sleep-wake circadian rhythm. However, reduced cortisol levels have been observed in both the urine and saliva of ME/CFS patients [16]. The low cortisol awakening response is closely associated with PEM and unrefreshing sleep in ME/CFS patients [17]. A gene profiling clinical study has demonstrated a significant correlation between glucocorticoid receptor (NR3C1) expression and the exacerbation of PEM symptoms in ME/CFS patients [18]. However, no animal-derived evidence verifying the ‘hyper-serotonergic hypothesis’ has been reported yet. In this study, fluoxetine (brand name: Prozac) was employed to implement hyper-serotonergic conditions, as it is a widely used selective serotonin reuptake inhibitor (SSRI) drug for treating depressive disorders. This drug is known to enhance 5-HT transmission by initially blocking the 5-HT transporter and subsequently desensitizing the 5-HT_1A_ receptor after 2 to 3 weeks [19].

Herein, we investigated the hyper-serotonergic hypothesis for the pathophysiology of ME/CFS through the utilization of a series of mouse models treated with a SSRI, a 5-HT synthesis inhibitor, and a viral vector for 5-HT_1A_ knockdown.

## Materials and methods

### Animals

The present study employed 384 specific pathogen-free C57BL/6J mice (9 weeks old, 352 males weighing 26–28 g, and 32 females weighing 18–20 g), obtained from Dae-Han Bio link Co., Ltd. (Eumseong, Rep. of Korea). The environmental room conditions were maintained at 23 ± 2 °C (temperature) and 55 ± 10% (humidity) using a thermohygrostat (ALFFIZ, BuSung Co., Ltd, Seoul, Rep. of Korea) under a 12-h light-dark cycle (09:00 to 21:00) with ad libitum feeding (Cargill Agri Furina, Gyeonggido, Rep. of Korea). Body weight and food intake were recorded during the experiments.

All experiments were approved by the Institutional Animal Care and Use Committee (IACUC) of Daejeon University (DJUARB2019-043) and were conducted in accordance with the Guide for the Care and Use of Laboratory Animals, as published by the National Institutes of Health (NIH).

### Eight serial animal experiments

We performed eight sequential experiments, namely the verification of ME/CFS-like behavior (Exp. 1 and 2), 5-HTergic hyperactivity (Exp. 3 and 4), hypothalamic–pituitary–adrenal (HPA)-axis response (Exp. 5 and 6), the reversal effect of a 5-HT synthesis inhibitor (Exp. 7) in mice injected with fluoxetine, and 5-HT_1A_ receptor knockdown (Exp. 8) using adeno-associated viral (AAV) vector injection into the dorsal raphe nucleus (DRN) (Additional file 1: Fig. S1A and B).

Briefly, after 1 week of acclimation, the mice were randomly allocated to the following experiments: Exp. 1 (n = 90 males and n = 32 females) for behavioral tests, immunofluorescence staining, and gene and protein expression analysis in model mice subjected to 4 weeks of fluoxetine injection; Exp. 2 (n = 16 males) to verify whether fatigue-like behavior recovers after a 6-week withdrawal from fluoxetine injection; Exp. 3 (n = 24 males) to determine the extracellular serotonin concentrations using microdialysis; Exp. 4 (n = 8 males) to investigate the 5-HT projections into the hypothalamus from the DRN; Exp. 5 (n = 112, males) to confirm the functional role of the 5-HT_1A_ receptor using an 8-OH-DPAT (5-HT_1A_ receptor full agonist) challenge; Exp. 6 (n = 32 males) to confirm response of the HPA axis to forced exercise; Exp. 7 (n = 10, males) to evaluate the reversal effect of the 5-HT synthesis inhibitor; and Exp. 8 (n = 60 males) to selectively validate the pathogenic role of 5-HTergic hyperactivity in the limbic region using a clustered regularly interspaced short palindromic repeats (CRISPR)-SaCas9-mediated Htr1a knockdown system. Fluoxetine hydrochloride (20 mg/kg) or normal saline for the control was intraperitoneally injected once daily for 28 days.

### Evaluation of ME/CFS-like symptomatic behaviors

A total of eight behavioral tests assessing fatigue-like behaviors (rota-rod test, fatigue rotating wheel test and home-cage activity test) and ME/CFS symptoms (nest building test for general malaise, plantar test for pain sensitivity, grip strength test for muscular strength, open field test for anxiety, and novel object recognition test for memory impairment) were conducted (Additional file 1: Fig. S1A, Exp. 1 and 2). All behaviors were recorded, and tracking data were calculated using a video camera connected to corresponding software (Smart Junior, Panlab SL, Barcelona, Spain). Performance was evaluated by researchers blinded to the experimental conditions. The detailed methods of the eight behavioral tests are described in the Additional file 1.

### Microdialysis for measuring extracellular 5-HT concentration

To measure extracellular 5-HT concentration, mice (under anesthesia with 2% isoflurane in 1 L/min oxygen) were implanted with a microinjection guide cannula (CMA 7; OD 0.58 mm; shaft length 5.0 mm) positioned 1 mm above the raphe nuclei (AP – 4.36, ML ± 0, DV – 3.0) using a robotic stereotactic drill and injection system (Neurostar, Tuebingen, Germany). The cannula was anchored with screws that were cemented with acrylic liquid. After 14 days of surgery, dialysate samples were collected using a microdialysis probe (CMA 7; OD 0.24 mm; shaft length 7.0 mm; membrane length 1.0 mm), syringe pump (CMA 402) and artificial cerebrospinal fluid (aCSF, 1 µL/min perfusion rate for 30 min). During dialysate collection, the mice were allowed to move freely in a Plexiglas cylinder cage (CMA 120), and the collected dialysates were stored in a refrigerated fraction collector (CMA 470) (Additional file 1: Fig. S1A, Exp. 3).

The extracellular concentration of 5-HT was analyzed with an LC-MS/MS method (SCIEX ExionLC system equipped with SCIEX triple quadrupole 6500+, CA, USA). Briefly, the dialysate was injected into a UHPLC with an Acquity UPLC HSS T3 column (1.8 μm, 2.1 × 100 mm), and the mobile phases were prepared as follows: (A) distilled water containing 0.1% formic acid and 5 mM ammonium formate and (B) acetonitrile/methanol (v/v, 1:1) containing 5 mM ammonium formate. The column was eluted at a flow rate of 0.3 mL/min with a gradient (5 to 90% B). Detection was performed using simultaneous pos/neg ion switching.

### Intracranial injection of retrograde tracer

One week before sacrifice, to trace the 5-HTergic projections to the hypothalamus from the DRN, fluoro-gold (FG, a retrograde fluorescent neuronal tracer, 2% in saline; 26,858, Cayman Chemicals, CO, USA), was injected into the left side of the hypothalamus at the following coordinates (relative to bregma): AP – 0.6, ML ± 0.2, DV – 4.8. An injection syringe (Hamilton, Nevada, USA) was used to deliver 200 nL of FG at a flow rate of 100 nL/min (Additional file 1: Fig. S1A, Exp. 4).

### Two challenge tests in measuring the HPA response

To evaluate the reactivity of the HPA axis and thermoregulation, mice were intraperitoneally injected with 0.1 mg/kg 8-hydroxy-2-dipropylamino tetralin hydrobromide (8-OH-DPAT, a 5-HT_1A_ receptor full agonist; H8520, Sigma, MO, USA). At 15 min intervals (from 0 to 45 min), plasma corticosterone levels were measured after sacrificing one experimental group, while another group was assessed for rectal temperature using a digital thermometer (BIO-TK8851, BIOSEB, France) (Additional file 1: Fig. S1A, Exp. 5).

To revalidate the HPA response to forcible exercise, mice were forced to run for 15 min at a speed of 10 rpm (3 m/min) using rotating wheel equipment, and then their plasma corticosterone levels were measured using an enzyme immunoassay kit (K014-H5, Arbor Assays, Michigan, USA) (Additional file 1: Fig. S1A, Exp. 6).

### Systemic injection of a 5-HT synthesis inhibitor

To confirm the pathological role of 5-HTergic hyperactivity, the reversible effects of a 5-HT synthesis inhibitor were measured by intraperitoneal injection of 4-chloro-dl-phenylalanine methyl ester hydrochloride (pCPA; HY-101456, MedChemExpress, NJ, USA) at a dose of 300 mg/kg twice daily for 3 consecutive days before behavioral tests (Additional file 1: Fig. S1A, Exp. 7).

### Construction viral vector for silencing 5-HT_1A_ receptor

To verify the pathogenic role of central 5-HT overactivation, we conducted a 5-HT_1A_ receptor gene (Htr1a) knockdown model using the clustered regularly interspaced short palindromic repeats (CRISPR)-SaCas9 system. Briefly, the pX601-AAV-CMV-SaCas9-hU6-single guide (sg) RNA plasmid was used (Additional file 1: Fig. S7A), and gRNA candidates for Htr1a and sham were selected using a CRISPR design tool (crispor.tefor.net). A mixture of Cas9 protein (100 ng/mL, EnGen Cas9 NLS; purchased from NEB) and gRNA (50 ng/mL) was injected into the perinuclear cytoplasm. The sgRNAs were generated with a MEGAshotscript T7 kit (Invitrogen, Carlsbad, CA, USA) using the sgRNA sequences listed in Additional file 1: Table S1. After exchanging the CMV for the human synapsin (hSyn) promoter, the sgRNAs were validated in two cell lines (Hepa1c1c7 hepatoma and Neuro2A neuroblastoma) using Lipofectamine 3000 reagents (Thermo, Waltham, MA, USA), as shown in Additional file 1: Fig. S7B to D. To titrate recombinant AAV vector particles, the plasmid vectors and packaging vector were co-transfected into AAV-293 cells using jetPEI (Polyplus-transfection, USA). At 3 days post-transfection, the virus was purified by ultracentrifugation with a CsCl density gradient, followed by quantification of virus titers using qPCR.

### Intra-DRN injection of viral vector

For viral vector injection into the DRN, mice were positioned in robotic stereotactic equipment (Neurostar, Tuebingen, Germany) under anesthesia with isoflurane (2% in 1 L/min oxygen). A hole was drilled over the DRN at the following coordinates (relative to bregma): AP – 4.36, ML ± 0, DV – 3.0. An injection syringe (Hamilton, Nevada, USA) was used to deliver 1 µL of virus (5 × 10^12^ genomic copies/mL) into the DRN at a flow rate of 0.1 µL/min. To minimize the upward flow of the viral solution, the needle was left in place for 8 min after injection. These mice were used for assessments of ME/CFS-like symptoms and plasma corticosterone levels (Additional file 1: Fig. S1B, Exp. 8).

### Blood and brain tissue collection

The day after the end of each experiment, the mice were sacrificed under CO_2_ anesthesia. Plasma was collected from abdominal blood by centrifugation at 3000×*g* for 15 min. After removing brain tissue immediately, each brain region was isolated using a coronal mouse brain matrix (1 mm, BSMAS001-1; Zivic Instruments, PA, USA) and biopsy punch (1 mm, BP-10 F, Kai Medical, Japan). For biochemical analysis, the brain tissue was homogenized in radioimmunoprecipitation assay (RIPA) buffer (R0278, Sigma, MO, USA) supplemented with protease and phosphatase inhibitor cocktails (#1861284, Thermo Scientific, MA, USA). For subcellular protein analysis, the cytoplasm, membrane and nucleus were extracted using a subcellular protein fractionation kit (#87790, Thermo Scientific, MA, USA). The total protein concentration was measured using a bicinchoninic acid protein assay kit (BCA1 and B9643, Sigma). For gene expression analysis, parts of the brain tissue were stored in RNAlater (Ambion, TX, USA). For immunohistological analysis, other mice were transcardially perfused with 0.05% heparin (10 units/mL in PBS) followed by 4% paraformaldehyde (PFA, pH 6.9), and then the brains were removed and placed in 4% PFA fixation solution.

### Plasma AST, ALT and TGF-β1 levels

Plasma levels of aspartate transaminase (AST) and alanine transaminase (ALT) were determined using an autoanalyzer (Chiron Diagnostics Co.). The transforming growth factor (TGF)-β1 active form in plasma was detected according to the manufacturer’s instructions (DY1679-05, R&D systems, Camarillo, USA).

### Immunofluorescence analysis

Immunofluorescence staining analysis for 5-HTergic activity was performed via 5-HT, 5-HT_1A_ receptor, FG and c-Fos staining in the raphe nuclei (RN). Immunofluorescence for glucocorticoid activity was performed via corticotropin-releasing hormone receptor (CRH_R) 2 and c-Fos staining in the paraventricular nucleus (PVN) of the hypothalamus. The brains were gradually cryoprotected in 10, 20 and 30% sucrose for 24 h each and were subsequently embedded in optimum cutting temperature (OCT) compound (Leica Microsystems, Bensheim, Germany) in liquid nitrogen. The embedded tissues were cut into frozen coronal section (35 μm) using a cryostat (CM3050_S, Leica) and then stored in free-floating buffer. After washing with ice-cold PBS, the sections were treated with blocking buffer (5% normal chicken serum in PBS and 0.3% Triton X-100) for 1 h at 4 °C and incubated with anti-goat 5-HT (1:200, ab66047, Abcam), anti-rabbit 5-HT_1A_ receptor (1:200, ab85615, Abcam), anti-rabbit FG (1:200, AB153, Millipore), anti-mouse c-Fos (1:1000, ab208942, Abcam), anti-rabbit CRH_R2 (1:200, NB100-56485, Novus), anti-rabbit Iba-1 (1:200, #019-19741, Wako), anti-rabbit glial fibrillary acidic protein (GFAP, 1:200, Z0334, Dako) and anti-goat doublecortin (DCX, 1:200, sc-8066, Santa Cruz) primary antibodies overnight at 4 °C. After washing with ice-cold PBS, the sections were incubated with a donkey anti-goat IgG H&L (1:400, Alexa Fluor® 488, ab150129), goat anti-mouse IgG H&L (1:400, Alexa Fluor® 488, ab150113), goat anti-rabbit IgG H&L (1:400, Alexa Fluor® 594, ab150080) or goat anti-mouse (1:400, Alexa Fluor® 594, ab150116) secondary antibody for 2 h at 4 °C. The sections were subsequently exposed to DAPI (1:1000, D9542, Sigma) to stain the cell nuclei. The signals were quantified using ImageJ 1.46 software (NIH, Bethesda, MD, USA).

### Western blot analysis

The protein expression levels of 5-HT_1A_ receptor, tryptophan hydroxylase (TPH) 2, c-Fos, CRH_R2, CRH, G protein-coupled inwardly-rectifying potassium channel (GIRK) 1, GIRK2, phospho-extracellular signal-regulated kinase (ERK)/ERK, phospho-cAMP response element-binding protein (CREB)/CREB, brain-derived neurotrophic factor (BDNF), vinculin, α-tubulin and β-actin in RN or hypothalamus homogenates were evaluated using western blotting analysis. After equalizing the protein concentrations, the homogenates were separated by 10% polyacrylamide gel electrophoresis and then transferred to polyvinylidene fluoride (PVDF) membranes. To minimize nonspecific binding, the membranes were blocked in 5% bovine serum albumin for 1 h. The membranes were incubated overnight at 4 °C with primary antibodies, including antibodies against the 5-HT_1A_ receptor (1:200, ab85615, Abcam), TPH2 (1:500, NB100-74555, Novus), c-Fos (1:1000, ab208942, Abcam), CRH_R2 (1:200, NB100-56485, Novus), CRH (1:500, 10944-1-AP, Proteintech), GIRK1 (1:100, MA5-25833, Invitrogen), GIRK2 (1:200, NB100-74575, Novus), p-ERK/ERK (1:200, #9101S/#9102S, Cell Signaling), p-CREB/CREB (1:200, ab32096/ab31387, Abcam), BDNF (1:200, ab108319, Abcam), vinculin (1:1000, sc-73614, Santa Cruz), α-tubulin (1:1000, ab7291, Abcam) and β-actin (1:1000, ab8229, Abcam). The membranes were incubated with an HRP-conjugated anti-rabbit, anti-mouse, or anti-goat antibody (GeneTex, Inc., Irvine, CA) for 1 h. The bands were visualized with an advanced enhanced chemiluminescence (ECL) kit. The intensity was analyzed by ImageJ version 1.46 (NIH, Bethesda, MD, USA).

### Real-time quantitative PCR analysis

The mRNA expression levels of the 5-HT_1A_ receptor and related transcriptional genes, including freud-1, deaf1, pet-1 and GAPDH, in the RN were analyzed by quantitative real-time PCR. Total RNA of samples was isolated using a RNeasy Mini Kit (Qiagen; Valencia, CA, USA), and cDNA was synthesized using a High-Capacity cDNA Reverse Transcription Kit (Ambion, Austin, TX, USA). Quantitative PCR was performed using SYBR Green PCR Master Mix (Applied Biosystems, Carlsbad, CA, USA), and PCR amplification was performed using a standard protocol with Rota-Gene Q real-time PCR (Qiagen; Valencia, CA, USA). Information regarding the primer sequences is summarized in Additional file 1: Table S2.

### ME/CFS patients and serum cortisol levels

To compare the cortisol levels between healthy controls and ME/CFS patients, we obtained serum samples from a cohort study (DJDSKH-22-BM-03). ME/CFS patients (between 18 and 65 years) were enrolled according to the diagnostic definition of IOM in the USA [1]. All subjects with other known causes of chronic fatigue were excluded, such as severe anemia, thyroid dysfunction and psychological disorders. Whole blood from 27 subjects (10 healthy and 17 patients) was collected between 10:00 and 11:30 am, and their serum cortisol levels were measured using an enzyme immunoassay kit (KGE008, R&D Systems, Minneapolis, MN).

### Statistical analysis

All results are expressed as the mean ± standard deviation (SD). Statistical significance was analyzed by unpaired *Student’s t*-test or one-way analysis of variance (ANOVA) followed by post hoc analysis by *Tukey’s t*-test using Prism 7 software (GraphPad). Differences at p < 0.05 were considered significant.

## Results

### Fatigue-like behaviors caused by 5-HTergic hyperactivity

**Fig. 1:**
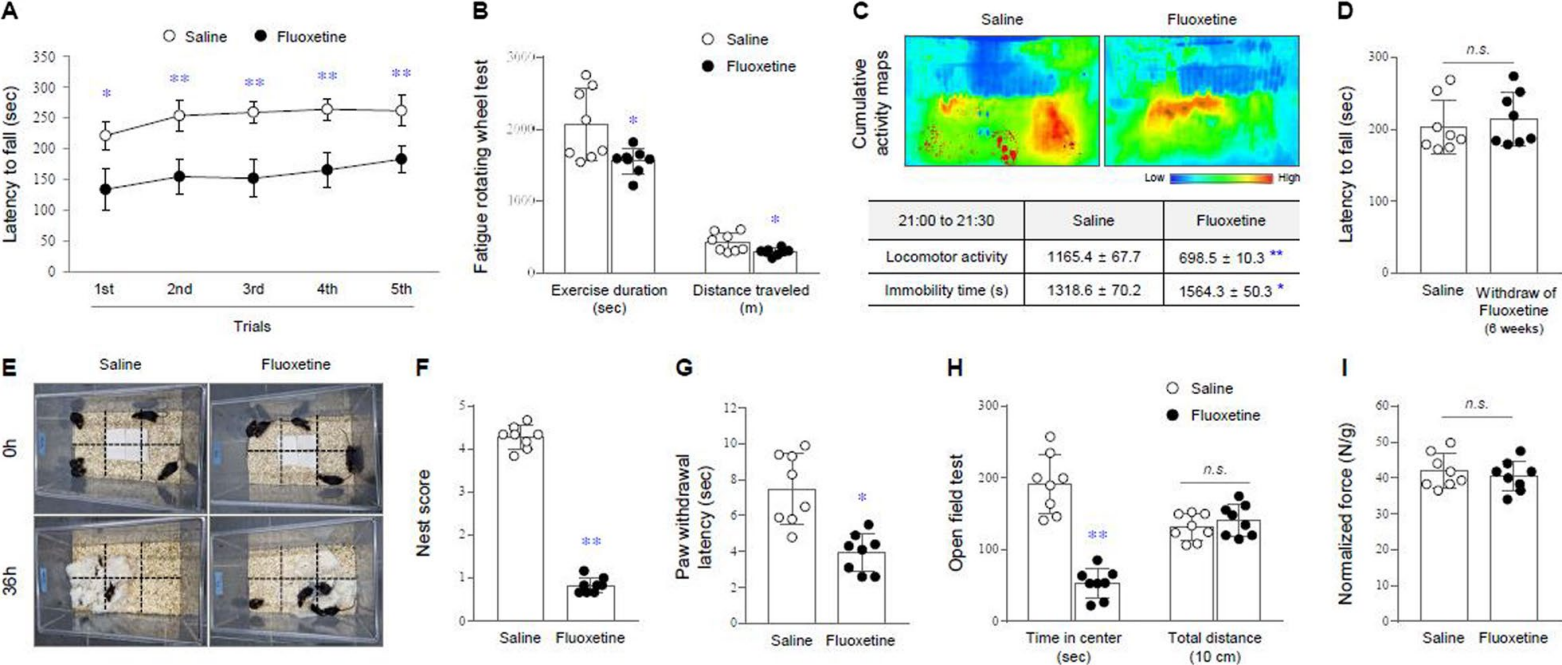
Fatigue- and ME/CFS-like behaviors. After fluoxetine injection for 4 weeks, fatigue-like behaviors were evaluated by both the latency to fall from the drum in rota-rod test (**A**) and the exercise duration and distance traveled in fatigue rotating wheel test (**B**). Locomotor activity and immobility duration for 1 h after awakening in the home-cage activity test (**C**) were assessed. The rota-rod test was then reperformed following 6 weeks of withdrawal of fluoxetine (**D**). Nesting score was determined according to the degrees to which the mice bit the squares, moved the squares into the corners and nested with the squares in the nest building test (**E** and **F**). The latency to paw withdrawal in the plantar test (**G**) and the time spent in the center and total distance traveled in the open field test (**H**) were assessed. Forelimb strength per body weight in the grip strength test (**I**) were assessed. The data are expressed as the mean ± SD (n = 5 or 8/group). *p < 0.05 and **p < 0.01 compared to the saline group

The 4-week fluoxetine injection induced fatigue-like behaviors compared with the saline group, as evidenced by a significant reduction in the latency to fall across five trials in the rota-rod test (p < 0.05 or 0.01, Fig. 1A) and decreases in exercise duration and traveled distance in the fatigue rotating wheel test (p < 0.05 for both parameters, Fig. 1B). Likewise, a significant reduction in locomotor activity and a notable increase in immobility time at their home-cage during the 30 min (21:00 to 21:30 pm) after waking up were also observed (p < 0.05 and 0.01, Fig. 1C). These results were similarly observed in female mice (Additional file 1: Fig. S2A and C). In addition, this fatigue-like behavior was abolished by the withdrawal of fluoxetine for 6 weeks (Fig. 1D).

### ME/CFS-symptoms caused by 5-HTergic hyperactivity

The chronic fluoxetine injection not only caused general malaise, as evidenced by the significant reduction in nest building scores (degree of biting squares, moving to corners, and nesting cotton during 36 h) (p < 0.01, Fig. 1E and F) but also increased pain sensitivity (rapid paw withdrawal from infrared irradiation) compared with the saline group (p < 0.05, Fig. 1G). For anxiety behaviors, the time spent in the center was significantly decreased in the fluoxetine-injected group compared with the saline group, but no significant differences in traveled distance were observed (p < 0.05, Fig. 1H).

On the other hand, fluoxetine injection tended to slightly decrease the strength-to-weight ratio in the grip strength test, but without statistical significance compared with the saline group (Fig. 1I and Additional file 1: Fig. S2B). The behavioral results for both nest building and grip strength test were observed in the same manner in female mice (Additional file 1: Fig. S2D and E).

### 5-HTergic hyper-activity and -projection in the limbic area

**Fig. 2:**
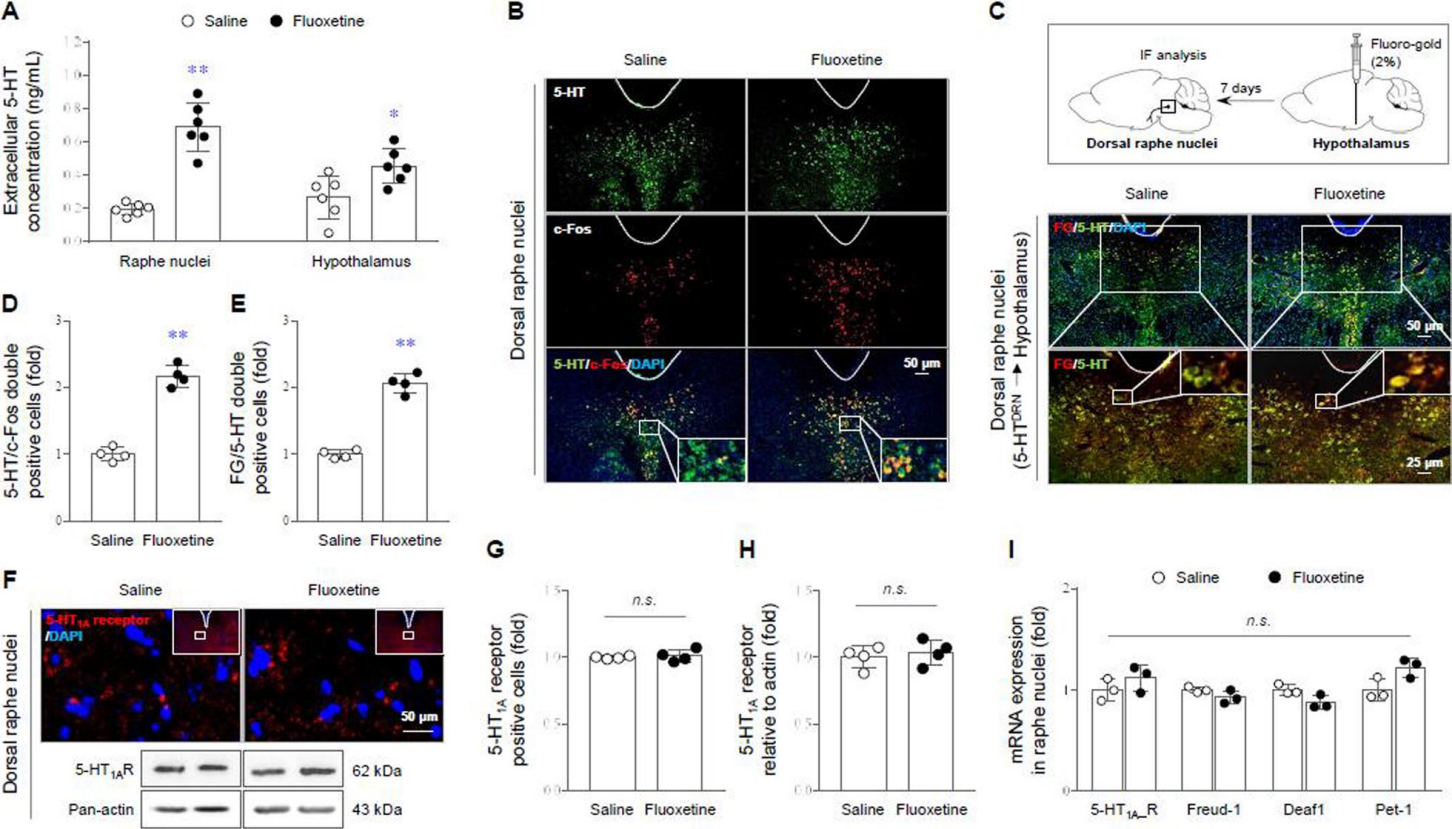
Serotonergic hyper-production and innervation. After fluoxetine injection for 4 weeks, mice were sacrificed. Serotonergic activity was evaluated by extracellular 5-HT concentrations in both the RN and hypothalamus using microdialysis and UHPLC analysis (**A**) and intracellular 5-HT/c-Fos double positive signals using immunofluorescence analysis in the DRN (**B** and **D**). The 5-HT projections into the hypothalamus were confirmed after 7 days injection of FG retrograde tracer, as shown in FG/5-HT double positive cells in DRN (**C** and **E**). The 5-HT_1A_ receptor-positive signal and 5-HT_1A_ receptor protein expression (**F**–**H**) in the DRN were evaluated. The mRNA levels of 5-HT_1A_ receptor transcriptional repressor and promotor genes were analyzed by real-time PCR (**I**). The data are expressed as the mean ± SD (n = 3, 4 or 6/group). *p < 0.05 and **p < 0.01 compared to the saline group

As expected, 4 weeks of fluoxetine injection considerably increased extracellular 5-HT concentrations in both regions of the DRN (over 3-fold, p < 0.01) and hypothalamus (approximately 2-fold, p < 0.05) (Fig. 2A) as well as significant increases of the intracellular 5-HT/c-Fos double-positive signal in the DRN (over 2-fold, p < 0.01, Fig. 2B and D). In addition, fluoxetine-induced notable increases in 5-HT^DRN→hypothalamus^ innervation was found by analysis of retrograde fluorescent tracer (FG)/5-HT-costained cells (p < 0.01, Fig. 2C and E).

### Functional loss of the 5-HT_1A_ receptor as a possible underlying mechanism

**Fig. 3:**
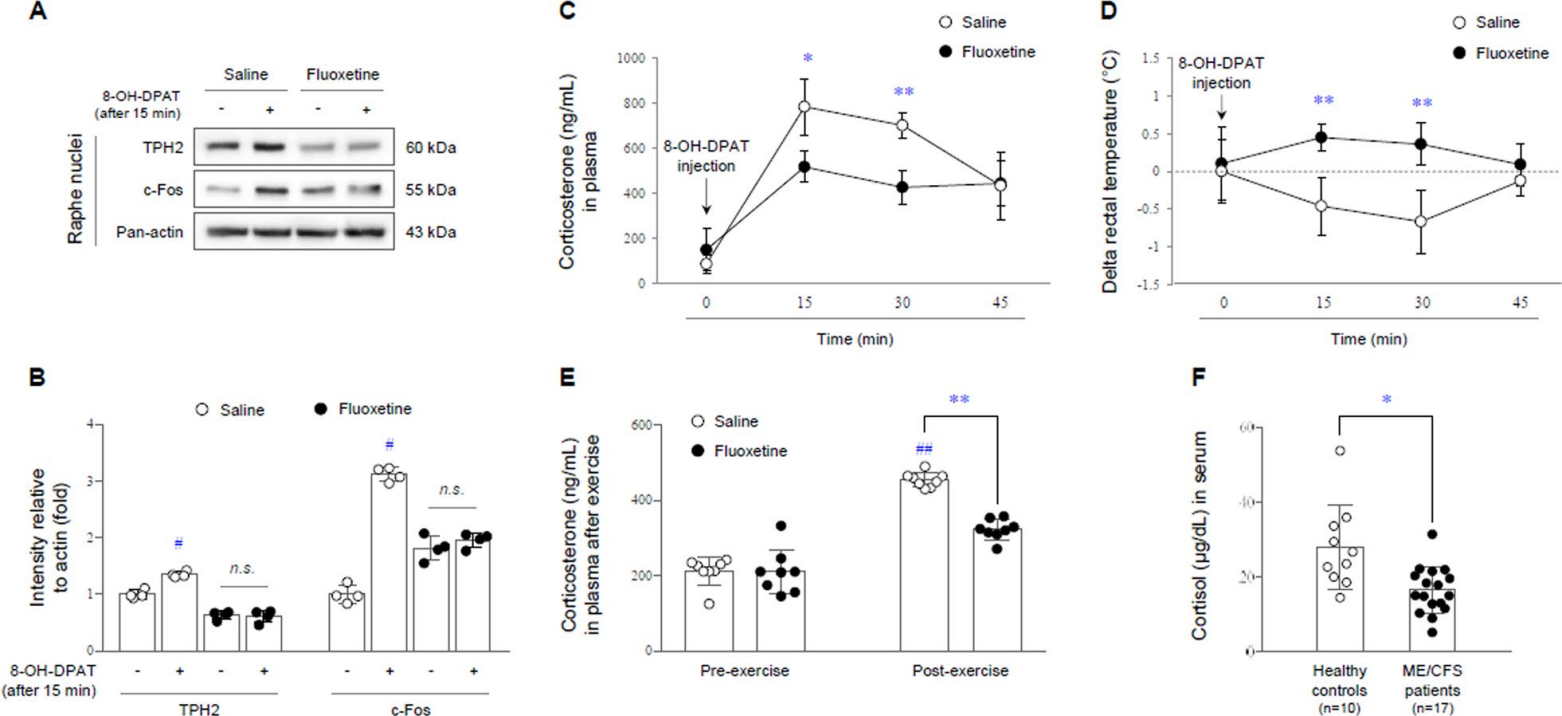
Functional loss of 5-HT_1A_receptor on the corticosterone and thermoregulatory responses. After fluoxetine injection for 4 weeks, the protein expression of TPH2 and c-Fos following 15 min of 8-OH-DPAT challenge was determined by western blot analysis in RN (**A** and **B**). Plasma corticosterone levels (**C**) or rectal temperature measurement (**D**) following 0, 15, 30, or 45 min of challenge with 8-OH-DPAT were analyzed. In addition, the plasma corticosterone levels were determined both pre- and post-exercise (**E**). The data are expressed as the mean ± SD (n = 4 or 8/group). *p < 0.05 and **p < 0.01 compared to the saline group, ^#^p < 0.05 and ^##^p < 0.01 compared to the saline-injected group without 8-OH-DPAT challenge or exercise. Serum cortisol levels of patients with ME/CFS were determined by EIA (**F**). The data are expressed as the mean ± SD (n = 10 and 17, respectively). *p < 0.05 compared to the healthy controls

The fluoxetine-evoked over-activations of 5-HT were not related to quantitative changes in the 5-HT_1A_ receptor expression, as evidenced by no changes in the protein and mRNA expression levels of the 5-HT_1A_ receptor as well as its transcriptional repressor (freud-1 and deaf1) and promoter (pet-1) genes (Fig. 2F–I). Nevertheless, 8-OH-DPAT (5-HT_1A_ receptor agonist) injection did not affect TPH2 and c-Fos protein expression (indicators of 5-HT neuronal activity after 15 min) in the DRN of the fluoxetine injected group, contrary to the saline group, (p < 0.05, Fig. 3A and B), evidencing functional loss of the 5-HT_1A_ receptor.

### Dysfunction of the HPA axis and hypothalamus

As expected, 8-OH-DPAT injection challenge rapidly increased the plasma corticosterone levels at after 15 and 30 min, while there was much less responsiveness in the fluoxetine-injected group (p < 0.05 or p < 0.01, Fig. 3C). This dysfunction of the HPA axis in the fluoxetine-adapted group was observed following the 15-min exercise challenge and was also correspondingly observed when comparing to pre-exercise conditions (p < 0.01, Fig. 3E).

**Fig. 4:**
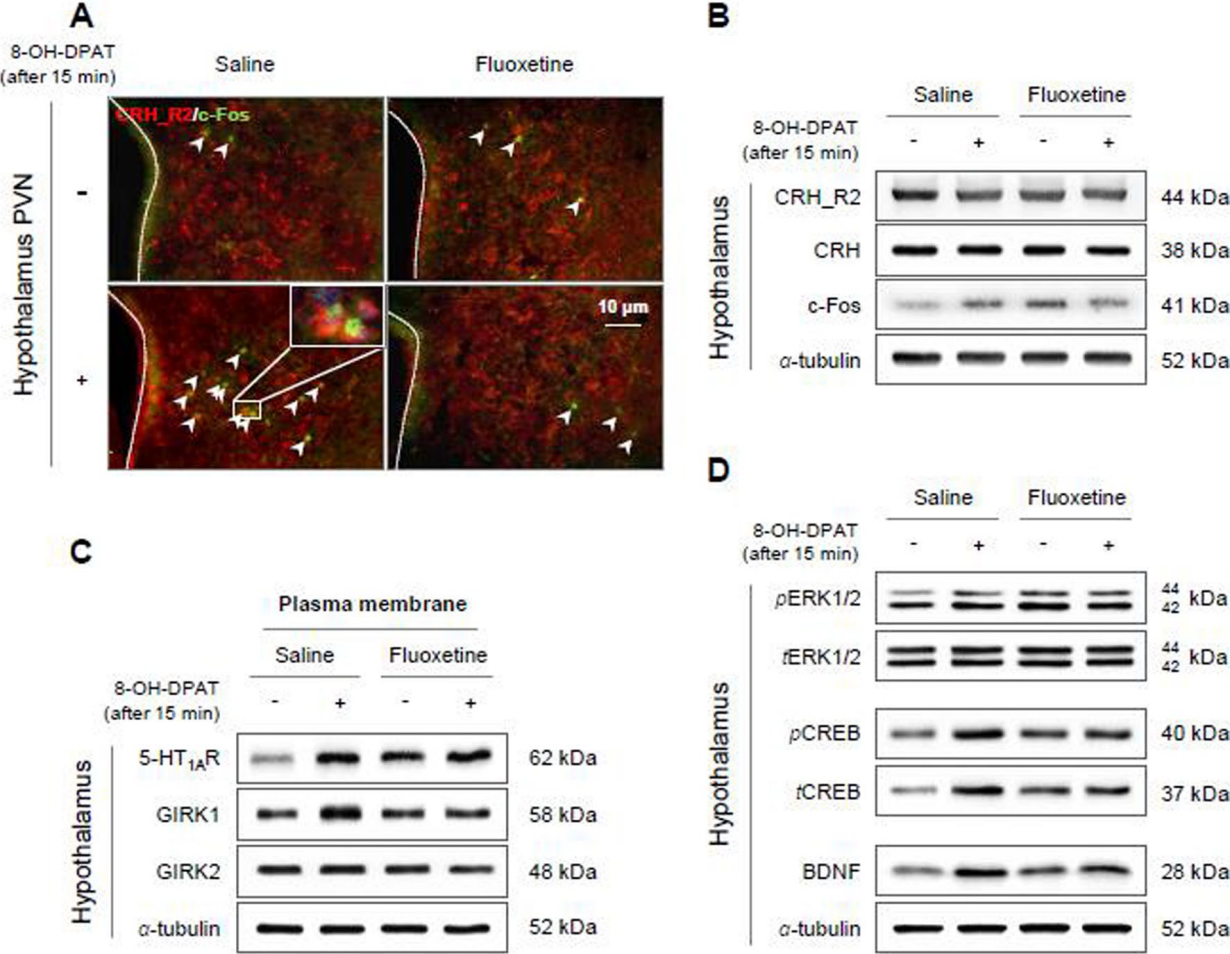
Hypothalamic neuronal activity under 5-HT_1A_receptor agonist treatment. After fluoxetine injection for 4 weeks, hypothalamic CRH neuronal activity (CRH, CRH_R2 and c-Fos) before and after challenge with 8-OH-DPAT was evaluated by immunofluorescent staining (**A**) and western blotting (**B**) analysis. In the hypothalamic plasma membrane, the protein expression levels of the 5-HT_1A_ receptors, GIRK1 and GIRK2 were measured (**C**). The protein expression levels of BDNF, phosphorylated ERK1/2 and CREB in whole hypothalamic lysate were analyzed (**D**). The data are expressed as the mean ± SD (n = 4/group)

In addition, the lowered rectal temperature by the 8-OH-DPAT challenge in the saline group was not observed in the fluoxetine-injected group (p < 0.01, Fig. 3D). This hypothalamic dysfunction in the fluoxetine-treated group was supported by the significantly lower response of CRH neurons (CRH receptor 2/c-Fos double-positive signals and CRH-related protein expressions), in contrast to the saline group (p < 0.05 or p < 0.01, Fig. 4A, B, and Additional file 1: Fig. S3A and B). The less response to 8-OH-DPAT in hypothalamic CRH neurons of fluoxetine-administered mice was re-confirmed by no changes in 5-HT_1A_ receptor and GIRK1 protein expression of the plasma membrane (but not for GIRK2) and its downstream molecules (phosphorylation of ERK1/2 and CREB and BDNF) compared to the saline group (p < 0.05 or p < 0.01, Fig. 4C, D, and Additional file 1: Fig. S3C–E).

We also found significantly lower levels of serum cortisol in 17 ME/CFS patients than in 10 healthy controls (p < 0.05, Fig. 3F).

### Validation of the pathological role of 5-HTergic hyperactivity

**Fig. 5:**
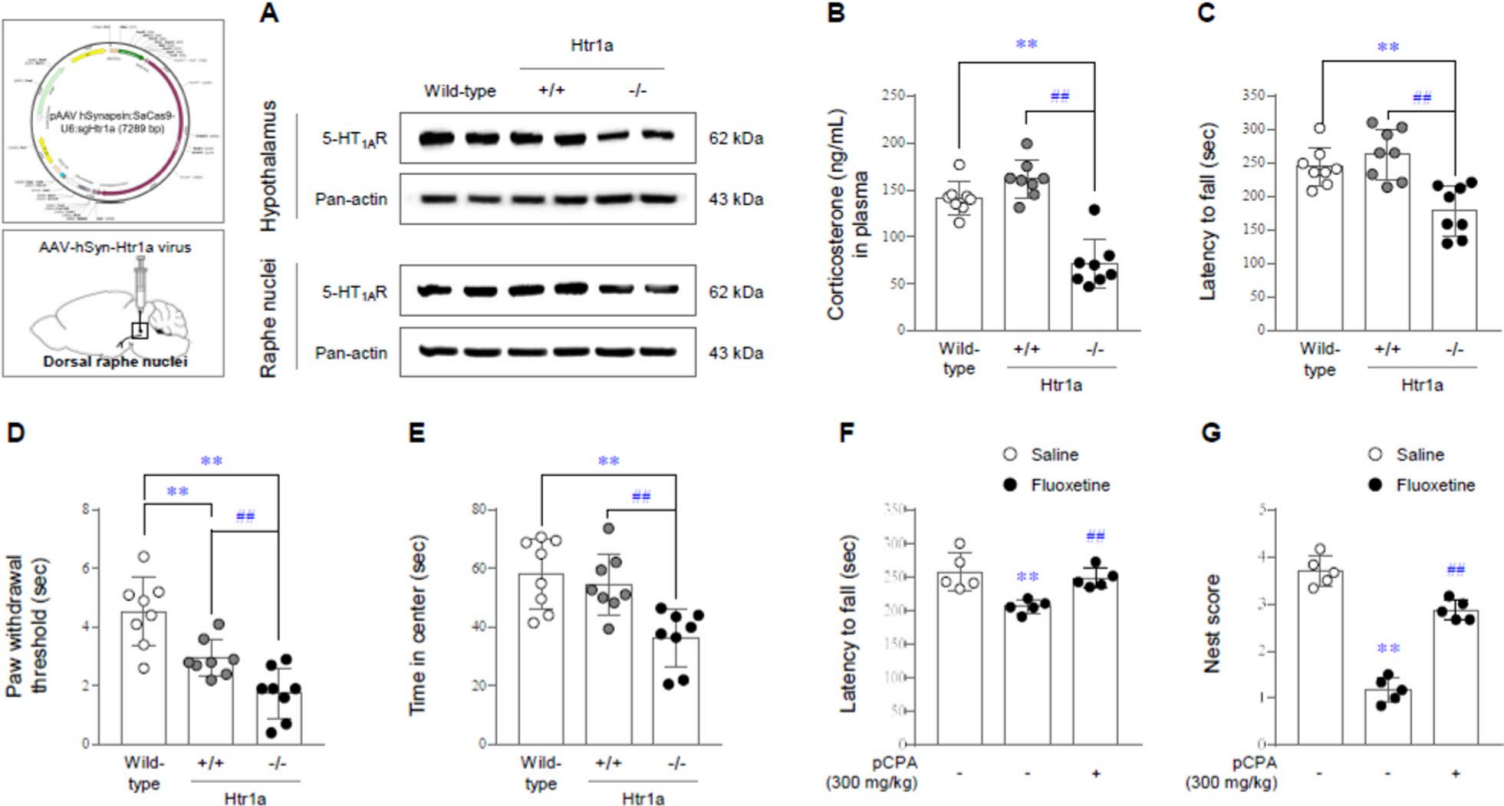
ME/CFS-like pathophysiology in Htr1a-/- mice and reverse effects of 5-HT synthesis inhibitor. In CRISPR/Cas9-mediated Htr1a-knockdown mice, the protein expression of the 5-HT_1A_ receptor in both the hypothalamus and RN was determined by western blot analysis (**A**). Plasma corticosterone levels in Htr1a-knockdown mice were assessed by EIA (**B**). The latency to fall from the drum in the rota-rod test (**C**), the latency to paw withdrawal in the plantar test (**D**) and the time spent in the center in the open field test (**E**) were assessed. Reverse effects of pCPA (5-HT synthesis inhibitor) against ME/CFS-like behaviors was evaluated by rota-rod and nest building test (**F** and **G**). The data are expressed as the mean ± SD (n = 4, 5 or 8/group). **p < 0.01 compared to the wild-type group or saline-injected group without pCPA treatment, ^#^p < 0.05 and ^##^p < 0.01 compared to the scrambled virus-infected group or fluoxetine-injected group without pCPA treatment

The pathological contribution of 5-HTergic hyperactivity was confirmed in two mouse models as follows. The Htr1a-targeting virus vector led to a notable knockdown of the 5-HT_1A_ receptor in both the RN and hypothalamus (p < 0.01 for both regions, Fig. 5A, and Additional file 1: Fig. S7E and F). These Htr1a^−/−^ mice showed significantly lower plasma corticosterone levels than wild-type or Htr1a^+/+^ mice (p < 0.01, Fig. 5B). Compared to wild-type or Htr1a^+/+^ mice, these mice also exhibited ME/CFS-related symptoms, including fatigue-like behavior in the rota-rod test (p < 0.01, Fig. 5C), augmented pain sensitivity in the plantar test (p < 0.01, Fig. 5D), and anxious behavior in the open-field test (p < 0.01, Fig. 5E).

Additionally, these fluoxetine-induced fatigue-like (rota-rod test) and ME/CFS symptomatic (nest building test) behaviors were considerably reversed by the administration of a 5-HT synthesis inhibitor (p < 0.01 for both behaviors, Fig. 5F and G).

## Discussion

Herein, we explored the central 5-HTergic hyperactivity hypothesis for ME/CFS pathogenesis based on previous clinical observations [13, 20, 21]. To investigate our hypothesis, we employed the chronic injection of fluoxetine, a representative SSRI (5-HT enhancer), and the dose for mice was set much higher than the clinical dose (> 2 to 5-fold). Furthermore, we employed the inbred strain C57BL/6J mouse because this was known to have a large Tph2 transcript expression and more 5-HT neurons than the BABL/c mouse [22]. SSRIs have been most frequently prescribed for the treatment of depression [23]; however, they are known to cause fatigue as a residual symptom [24–26]. As expected, a 4-week injection of fluoxetine into mice elicited ME/CFS-like behaviors, which were abolished after 6 weeks of withdrawal (Fig. 1A, B and D). In fact, two groups reported that healthy athletes showed rapid exhaustion and fatigue following exercise by the administration of SSRIs [27, 28]. A recent cohort study also found that SSRI medications lowered the overall physical movement of 266 participants under the accelerometer test compared with non-SSRI controls [29].

We found notably lower locomotor activity during the 30 min after awakening in the fluoxetine-injected group (Fig. 1C and Additional files 2, 3, 4, 5), reflecting unrefreshing sleep, one of the two core symptoms present in over 90% of ME/CFS [30]. Along with PEM, unrefreshed sleep is considered the best distinguishable diagnostic predictor between ME/CFS and other fatigue-accompanied disorders, such as idiopathic chronic fatigue and depressive disorders [31]. Our mouse model also showed general malaise and flu-like symptoms as well as pain exacerbation and anxiety behavior (Fig. 1E–H), which are complained by approximately 30 to 70% of patients with ME/CFS [32–34]. In contrast, no significant muscular weakness was observed (Fig. 1I), supporting brain-specific disturbances rather than physical fatigability as a component of ME/CFS pathophysiology [35].

Due to the debilitating symptoms described above, more than 25% of patients with ME/CFS are housebound or bedbound, and half of patients are unable to work full time [36]. Among various suggestions to explain the pathophysiology of ME/CFS, 5-HTergic hyperactivity is a considerable hypothesis. The brain serotonin hyperactivity of ME/CFS patients was observed from neuroimaging PET data showing reduced levels of the serotonin transporter (a 5-HT reuptake molecule) in the anterior cingulate area [12] and the 5-HT_1A_ receptor (a negative feedback loop) in multiple brain regions [11]. Two other clinical datasets also revealed that ME/CFS patients had 1.5-fold increase in free tryptophan (5-HT precursor) availability to the brain [37] and a reduction in the central serotonin metabolic rate [38]. To obtain empirical evidence for the above clinical observations, we first confirmed the ME/CFS-like features under hyperserotonergic manipulation (extra- and intracellular hyperactivity of 5-HT) in the RN along with high 5-HT^DRN→hypothalamus^ innervation (Fig. 2A–E).

In the past, certain physicians tended to consider ME/CFS a psychiatric disorder rather than a real physical disease [39], thus, many medical doctors have prescribed antidepressants to patients with ME/CFS given the related psychological issues [33]. Several clinical trials however, found that antidepressants, including SSRIs, were ineffective against ME/CFS and occasionally even caused patients to feel further fatigue [40, 41]. 5-HT is a representative neurotransmitter that in involved in emotional control, especially satisfaction and happiness. Unbalanced 5-HT activity causes various disorders, such as depression in the case of low activity but central fatigue in the case of excess [42]. Accordingly, the synthesis and turnover of 5-HT are rigidly controlled, especially in the brain, and the 5-HT_1A_ receptor is most well-known as a key player performing negative feedback [43]. Contrary to the hyperactivity of the 5-HT_1A_ receptor in depressive disorder [44], the present study revealed the loss of function of the 5-HT_1A_ receptor, leading to 5-HTergic hyperactivity in the limbic area (Figs. 2F–I and 3A and B).

Among several limbic areas, the hypothalamus is one of the largest 5-HT projecting regions [45]. Although 5-HT transmission into the hypothalamus is necessary to maintain the HPA endocrine axis and autonomic nerve system [46], its excessive projection leads to hypothalamic dysfunction through desensitized 5-HT_1A_ receptor [47, 48]. Our data presented 5-HTergic hyperactivity-mediated hypothalamic dysfunction, as shown by the blunted HPA axis and thermoregulatory response to a 5-HT_1A_ receptor agonist or exercise challenge (Fig. 3C–E). In fact, dysfunction of the HPA axis is one of the most well-known clinical features of ME/CFS [49]. Therefore, the 5-HT_1A_ receptor agonist (buspirone or ipsapirone) challenge test has been used for the diagnosis of ME/CFS via measurement of prolactin or adrenocorticotropic hormone (ACTH) released from the hypothalamus [14, 50]. The hypothalamus is known as a master gland that orchestrates several vital functions, such as the sleep-wake rhythm, thermoregulation, appetite-energy balance, and emotional and stress responses [51]. As we predicted, our mouse model showed the above multiple dysfunctions in the morning awakening response (Fig. 1C), rectal temperature response (Fig. 3D), corticosterone response (Fig. 3C and E) and anxiety (Fig. 1H).

A weak response by the HPA axis could explain at least hypocortisolism and PEM symptoms [17]. Recent fMRI studies of patients with ME/CFS have indicated impairment of reciprocal connectivity among several brain regions including the hypothalamus [52], and exercise-mediated overactivation in the whole RN and midbrain [53]. Consistent with reports of a reduced salivary cortisol awakening response and reduced serum ACTH levels [54], we also observed significantly reduced serum levels of cortisol in seventeen patients with ME/CFS (Fig. 3F). This hypocortisolism was caused by an impaired CRH neuronal response to a 5-HT_1A_ receptor agonist in our hyperserotonergic animal model (Fig. 4A–D and Additional file 1: Fig. S3). Previous studies also supported our results, as indicated by the long-term SSRI-induced low cortisol responses in the offspring of both zebrafish [55] and pregnant women [56]. Most importantly, we further validated the above findings by using CRISPR-mediated 5-HT_1A_ receptor knockdown system and 5-HT synthesis inhibitor (Fig. 5A–G and Additional file 1: Fig. S2F).

**Fig. 6:**
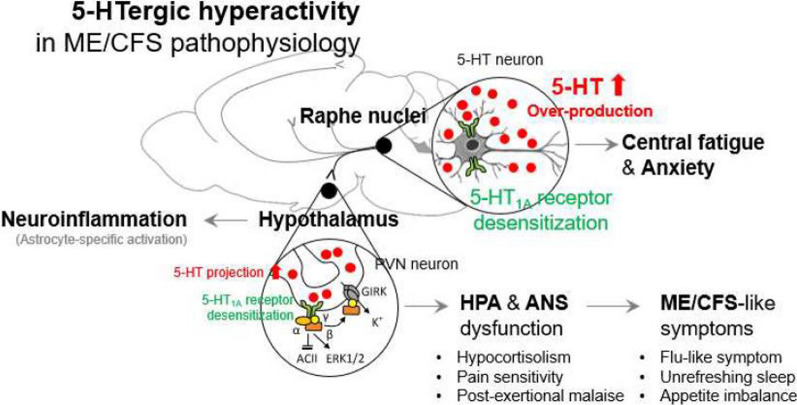
Pathophysiological mechanisms of 5-HTergic hyperactivity in ME/CFS. High 5-HTergic innervation causes central fatigue and ME/CFS-representative features, including dysfunction of hypothalamic–pituitary–adrenal (HPA) and autonomic nervous system (ANS) via desensitization of 5-HT_1A_ receptor

Herein, we provide animal evidence supporting the 5-HTergic hyperactivity hypothesis for ME/CFS pathophysiology, as summarized in Fig. 6. Our findings were also reproduced in female mice (Additional file 1: Fig. S2A–E). As an important clinical signature in ME/CFS patients regarding an elevated serum level of TGF-β1 reflecting the severity of fatigue [8, 57], we also found high level of plasma TGF-β1 in both models by chronic fluoxetine injection and 5-HT_1A_ receptor-knockdown (Additional file 1: Fig. S4E and F). Some data have revealed certain levels of neuroinflammation in the brains of ME/CFS patients [58], which was confirmed by the elevations in reactive astrocytes (but without changes in microglia) (Additional file 1: Fig. S4A–D). Although there may be a gap between mouse models and humans, the present study is a timely step.

Either orthostatic intolerance or cognitive impairment is required as one of the optional symptoms for the IOM diagnostic criteria [30]. Both the reduced wheel-balance activity in the rota-rod test and the reduced HPA axis response indicate orthostatic intolerance (Figs. 1A and 3C to E). One limitation of the present study was that there was no alteration in cognitive function (Additional file 1: Fig. S6A and B). Notably, fluoxetine does not desensitize the 5-HT_1A_ receptor in the hippocampus [59], and it conversely enhances hippocampal neurogenesis, as described previously [60]. In clinical practice, fluoxetine can even cause anorexia on rare occasions [61], while our animal model showed an increasing pattern of food intake (only in males) but slightly lowered body weight in both sexes (Additional file 1: Fig. S5A to D). No influence on hepatic enzymes was observed after 4 weeks of fluoxetine injection (Additional file 1: Fig. S5E). Along with these limitations, our hypothesis needs to be reaffirmed in other animals or by pathogenic conditions, and our findings have limitations that do not fully reflect the genetic, environmental, and immunological complexities of ME/CFS patients.

Overall, our study provides the first translational evidence for the involvement of 5-HTergic hyperactivity in the pathophysiology of ME/CFS. With our novel animal model for ME/CFS, this study helps to clarify one of the potent pathophysiological mechanisms of this syndrome. In addition, we provide diagnostic clues (high central serotonin) that can be used to differentiate ME/CFS from similar diseases, such as fibromyalgia and depressive disorders.

### Supplementary Information

**Additional file 1. **Supplementary Materials. **Fig. S1.** Experimental designs. **Fig. S2.** Fatigue and ME/CFS-like behaviors in female mice. **Fig. S3.** Quantifications of hypothalamic protein expression. **Fig. S4.** Microglial and astrocytic activity and plasma TGF-β1 levels. **Fig. S5.** Body weight, food intake, and the levels of liver enzymes. **Fig. S6.** Cognition and hippocampal neurogenesis. **Fig. S7.** CRISPR/Cas9-mediated Htr1a knockdown. **Table S1.** Target sequence for Mus musculus Htr1a. **Table S2.** Sequence of the primers used in real-time PCR analysis.

**Additional file 2.** Video for home-cage activity (Saline-injected male mice).

**Additional file 3.** Video for home-cage activity (Fluoxetine-injected male mice).

**Additional file 4.** Video for home-cage activity (Saline-injected female mice).

**Additional file 5.** Video for home-cage activity (Fluoxetine-injected female mice).

## Data Availability

The datasets used and/or analyzed during the current study are available from the corresponding author on reasonable request.

## Abbreviations

ACTH: Adrenocorticotropic hormone
ALT: Alanine transaminase
AST: Aspartate transaminase
BDNF: Brain-derived neurotrophic factor
CREB: cAMP response element-binding protein
CRH: Corticotropin-releasing hormone
DCX: Doublecortin
DRN: Dorsal raphe nucleus
ERK: Extracellular signal-regulated kinase
FG: Fluorogold
GFAP: Glial fibrillary acidic protein
GIRK: G protein-coupled inwardly rectifying potassium channel
HPA: Hypothalamic–pituitary–adrenal
5-HT: 5-Hydroxytryptamine
ME/CFS: Myalgic encephalomyelitis/chronic fatigue syndrome
8-OH-DPAT: 8-Hydroxy-2-(dipropylamino)tetralin hydrobromide
pCPA: 4-Chloro-dl-phenylalanine methyl ester hydrochloride
PEM: Postexertional malaise
PVN: Paraventricular nucleus
SSRI: Selective serotonin reuptake inhibitor
TGF: Transforming growth factor
TPH: Rryptophan hydroxylase
