# Supplementary material for: Central 5-HTergic hyperactivity induces myalgic encephalomyelitis/chronic fatigue syndrome (ME/CFS)-like pathophysiology
Source: J Transl Med. 2024 Jan 8;22:34. doi: 10.1186/s12967-023-04808-x (PMC10773012; doi:10.1186/s12967-023-04808-x)

**Additional file**

**Materials and Methods**

**Behavioral tests**

**Rota-rod test**

The evaluation of motor activity was performed using a rota-rod machine (ENV-574M, Med Associates Inc., VT, USA) according to the manufacturer’s instructions. Briefly, the mice were habituated on the stationary drum at 4 rpm for 60 sec and subsequently at an accelerating speed from 2 to 20 rpm for 500 sec. During habituation, the mice were immediately placed back on the drum at most five times after they fell to the bottom of the apparatus. The motor activity and sense of balance were evaluated by the latency to fall during drum acceleration from 4 to 40 rpm for 300 sec (5 trials interval 20 min), respectively.

**Fatigue rotating wheel test**

To evaluate fatiguability, exercise capacity was measured using motor-driven rotating wheel equipment (YLS-B10, Yiyuan Technology Development Co., Shandong, China) according to the manufacturer’s instructions with slight modification. Briefly, the exercise procedure involved a gradual increase in velocity from 15 to 30 rpm and an electric shock of 2.0 mA. If a mouse kept giving up running for over 2.5 sec even with electric shocks, the rotating wheel was stopped for 30 sec. Three time-repeated stopping for 30 sec was considered to be exhibiting physical exhaustion of mice. Exercise capacity was evaluated by two parameters (exercise duration and distance traveled).

**Home-cage activity test**

To analyze the behaviors upon awakening, the locomotor activity in the home cage (length 30 × width 20 × height 12 cm) was monitored using an infrared LED-HD camera (DrivePro-Body 30, Transcend Information Inc., Taipei, Taiwan). Video recordings from 9:00 to 9:30 pm were analyzed using software (Smart 3.0, Panlab SL, Barcelona, Spain). Awakening-activity was evaluated by two parameters (global activity and immobility time).

**Nest building test**

The evaluation of general malaise behavior was conducted using nest building score. Briefly, a total of 12 g of pressed cotton squares (7 × 5 cm, Envigo, IN, USA) were placed in the floor center of a cage housing five mice. The degrees to which the mice bit the squares, moved them into the corners and nested with the squares overnight were scored from 0 to 5.

**Plantar test**

Pain sensitivity was determined using a Hargreaves apparatus (37370-002, Ugo Basile, Comerio, Italy) according to the manufacturer’s instructions. Briefly, each mouse was placed in a plexiglass cubicle (length 8.5 × width 3.4 × height 3.4 cm) for 15 min of habituation. A constant-intensity radiant heat source (intensity 70 I.R.) was focused on the mice plantar surface of the hind paw. The latency to paw withdrawal was recorded.

**Grip strength test**

The evaluation of muscular strength was performed using a grip strength test apparatus (BIO-G53, BIOSEB, FL, USA) connected to a wire grid (length 15 × width 9 cm) and an isometric force transducer. Briefly, mice were allowed to grasp the grid with their fore paw and gently pulled backward until they lost grip within 3 sec. This process was repeated for three times at interval 5 min, and mean maximal force is expressed in Newtons (N).

**Open field test**

To evaluate anxious behavior, a large square chamber (length 40 × width 40 × height 30 cm) was used. Briefly, the center area (25 × 25 cm) was designated in recording software. After acclimation for 30 min in a testing room under 50 lux illumination, the mouse was allowed to freely explore the field for 5 min. The time spent in the center area was recorded.

**Novel object recognition test**

The evaluation of recognition memory was conducted using a visual cue on one side of open box (length 40 × width 40 × height 30 cm) and two identical objects. Briefly, mice were allowed to freely explore the box for 8 min without objects (habituation session), and after 12 h, each mouse was placed for 10 min in the center of an open box with two identical objects placed on opposite corners (training session). Six hours later, exploring behaviors were recorded for 10 min in the presence of one familiar and one novel object (test session). Exploration was defined as directing the mouse’ nose toward the novel object closely within 2 cm. Discrimination index was calculated as follows: [time spent exploring novel object/(time spent exploring familiar and novel objects)]


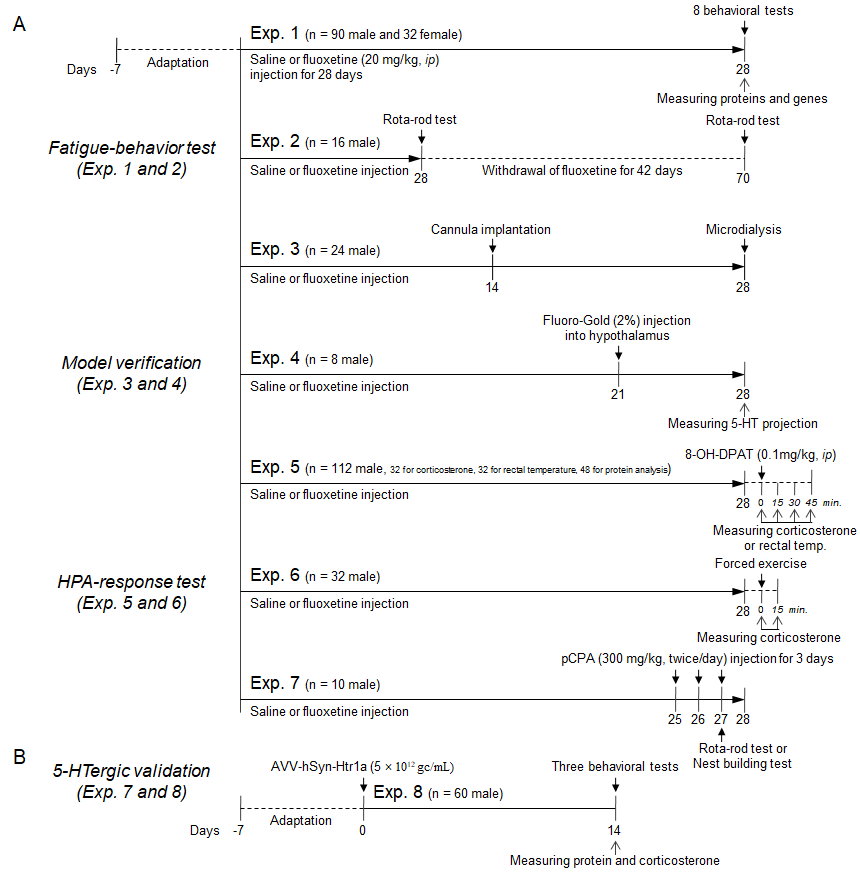


**Figure S1. Experimental designs.** Eight serial experiments were conducted as follows. Experiments for evaluating behavioral tests, protein and gene expression analyses, serum biochemistry and immunohistology analyses (Exp. 1) and abolishment of fatigue-like behavior following withdrawal of fluoxetine (Exp. 2) were performed. Experiments for measuring extracellular serotonergic concentration (Exp. 3) and serotonergic innervation (Exp. 4) were performed to verify the ME/CFS-like model reflecting serotonergic hyperactivation. The corticosterone and/or thermoregulatory responses following 8-OH-DPAT challenge (Exp. 5) or forced exercise (Exp. 6) were observed. Using a 5-HT synthesis inhibitor (Exp. 7) and CRISPR/Cas9-mediated Htr1a knockdown system (Exp. 8), the ME/CFS pathophysiological features were validated (A and B).


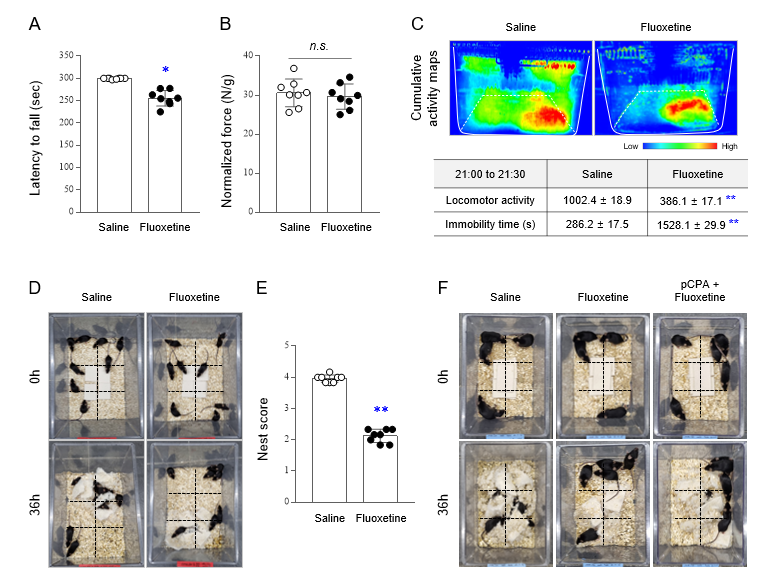


**Figure S2. Fatigue and ME/CFS-like behaviors in female mice.** The latency to fall from the drum in the rotarod test (A); the forelimb strength per body weight in the grip strength test (B); the locomotor activity and immobility duration for 1 h after the mice awakened in the home-cage activity test (C); and the nesting scores, which were assigned according to the degrees to which the mice bit the squares, moved the squares into the corners and nested with the squares in the nest building test (D and E) were assessed in female mice. Nest building behaviors of male mice subjected to injection of pCPA (5-HT synthesis inhibitor) were evaluated (F). The data are expressed as the mean ± SD (n = 5 or 8/group). *p < 0.05 and **p < 0.01 compared to the saline-injected group.


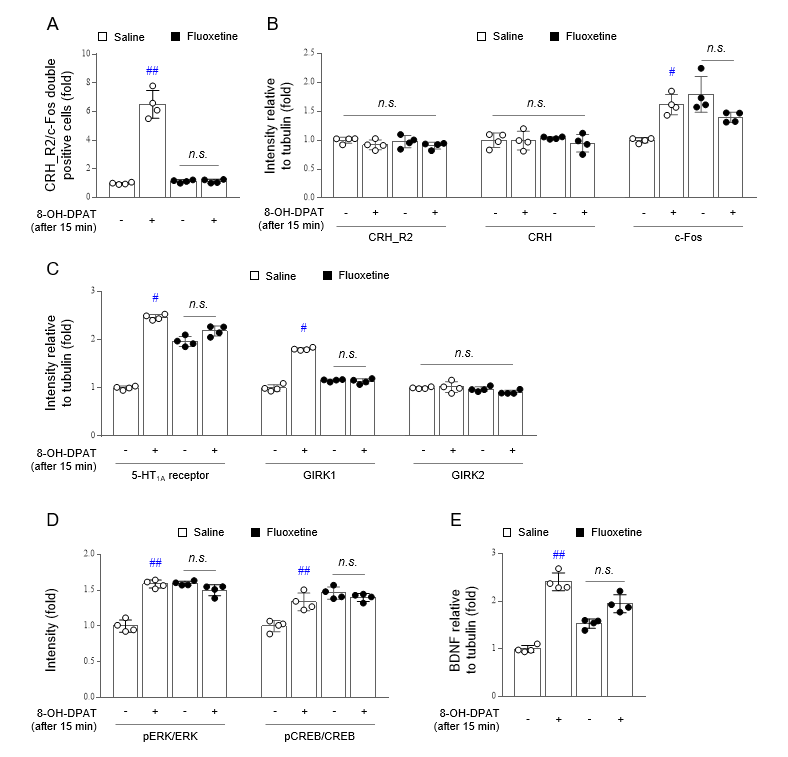


**Figure S3. Quantifications of hypothalamic protein expression.** Following 15 min of 8-OH-DPAT challenge, the CRH_R2/c-Fos double-positive signals in the hypothalamic PVN were semiquantified (A). Under same condition, the protein expression of CRH_R2, CRH and c-Fos in whole hypothalamic lysate (B) and 5-HT_1A_ receptor, GIRK 1 and GIRK2 in hypothalamic plasma membrane (C) and phosphorylated ERK/total ERK, phosphorylated CREB/total CREB and BDNF in whole hypothalamic lysate (D and E) were semiquantified. The data are expressed as the mean ± SD (n = 4/group). #p < 0.05 and ##p < 0.01 compared to the saline-injected group without 8-OH-DPAT challenge.


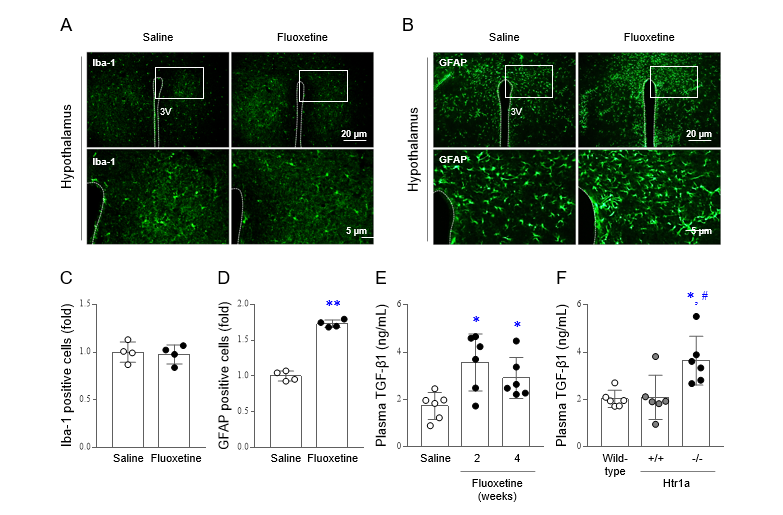


**Figure S4. Microglial and astrocytic activity and plasma TGF-β1 levels.** The Iba-1 (A) and GFAP (B) positive signals in the hypothalamus were evaluated, and their intensity was semiquantified (C and D). Plasma TGF-β1 levels were determined in fluoxetine-exposed (E) and Htr1a-knockdown group (F). The data are expressed as the mean ± SD (n = 4 or 6/group). *p < 0.05 and **p < 0.01 compared to the saline-injected group or wild-type group, #p < 0.05 compared to the scrambled virus-infected group.


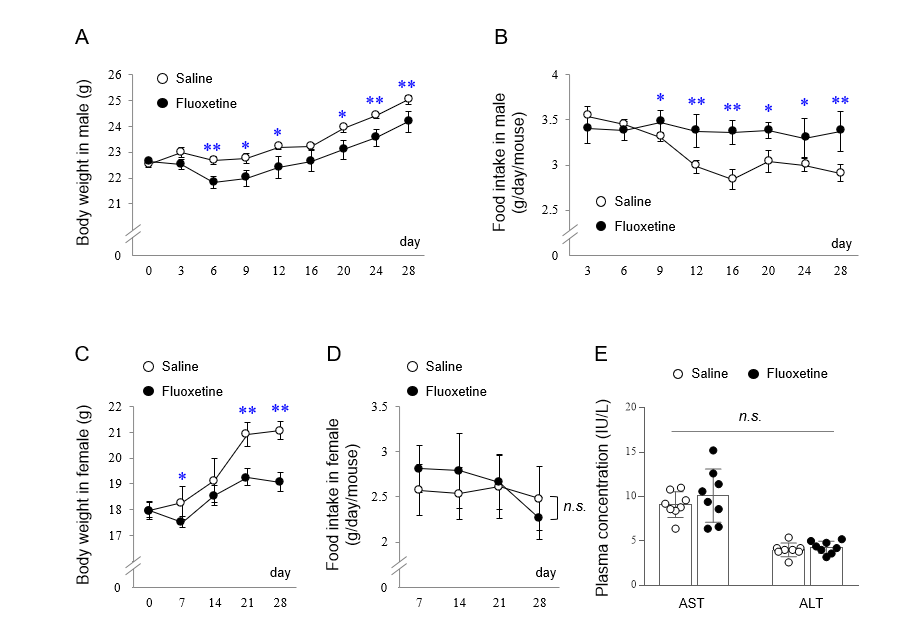


**Figure S5. Body weight, food intake, and the levels of liver enzymes.** During the experimental days, the body weight and food intake in male (A and B) and in female (C and D) were recorded. The influences of fluoxetine on the levels of plasma hepatic enzymes, including AST and ALT (E) were determined. The data are expressed as the mean ± SD (n = 5 or 8/group). *p < 0.05 and **p < 0.01 compared to the saline-injected group.


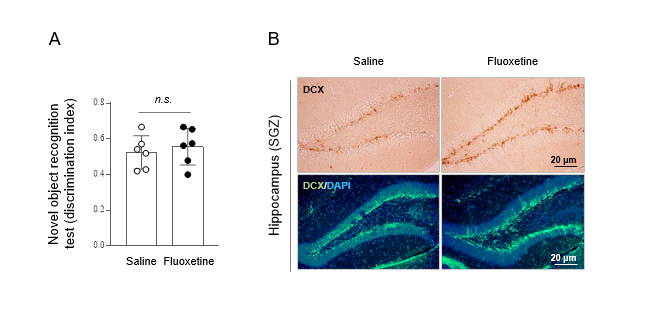


**Figure S6. Cognition and hippocampal neurogenesis.** The discrimination index in the novel object recognition test (A) and hippocampal neurogenesis (DCX-positive signal) were analyzed (B). The data are expressed as the mean ± SD (n = 3 or 6/group).


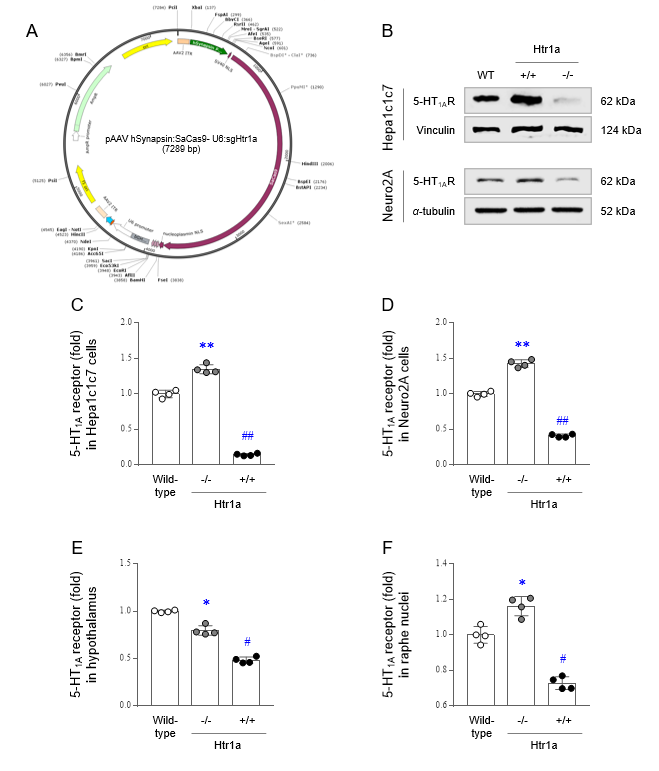


**Figure S7. CRISPR/Cas9-mediated Htr1a knockdown.** An AAV vector map was designed (A). Validation of Htr1a knockdown was performed using two murine cell lines (B), Hepa1c1c7 (C) and Neuro2A (D), and two target brain areas, the hypothalamus and RN (E and F). The data are expressed as the mean ± SD (n = 4/group). *p < 0.05 and **p < 0.01 compared to the wild-type cells or mice, #p < 0.05 and ##p < 0.01 compared to the scrambled virus-infected cells or mice.

**Table S1.** Target sequence for Mus musculus Htr1a.

| sgHtr1a | Target sequence | PAM | GC% |
| --- | --- | --- | --- |
| +/+ | GAGGACCAGCATGAGCAGCAG | CGGAAT | 61.90 |
| -/- | GAGTGCACCATCAGCAAGGACC | ACGGGT | 59.09 |

PAM; protospacer adjacent motif, GC; guanine and cytosine.

**Table S2.** Sequence of the primers used in real-time PCR analysis.

| Gene (number) | Primer sequencing (Forward and Reverse) |
| --- | --- |
| Htr1a  (NM_008308.4) | 5′-CCC CAA CGA GTG CAC CAT-3′  5′-GCG CCG AAA GTG TAG AT-3′ |
| Freud-1  (NM_145970.2) | 5′-GCT GGC AGA GCT AAA TGA GGT-3′  5′-GTG CAG ACT GGT AGA GGG TTA-3′ |
| Deaf1  (NM_001282076.1) | 5'-GTG TAT CAA GCA GGG AGA AAA C-3'  5'-CGG ATG CTT CTC TTC CAG TC-3 |
| Pet-1  (NM_153111.2) | 5'-GCG ACT TGG GGG GTC ATT ATC AC-3'  5'-GCC TGA TGT TCA AGG AAG ACC TCG G-3' |
| GAPDH  (NM_001289726) | 5'-ACA TCA TCC CTG CAT CCA CT-3'  5'-AGA TCC ACG ACG GAC ACA TT-3' |

Htr; 5-Hydroxytryptamine receptor, GAPDH; Glyceraldehyde-3-phosphate dehydrogenase

**Full-length blots**


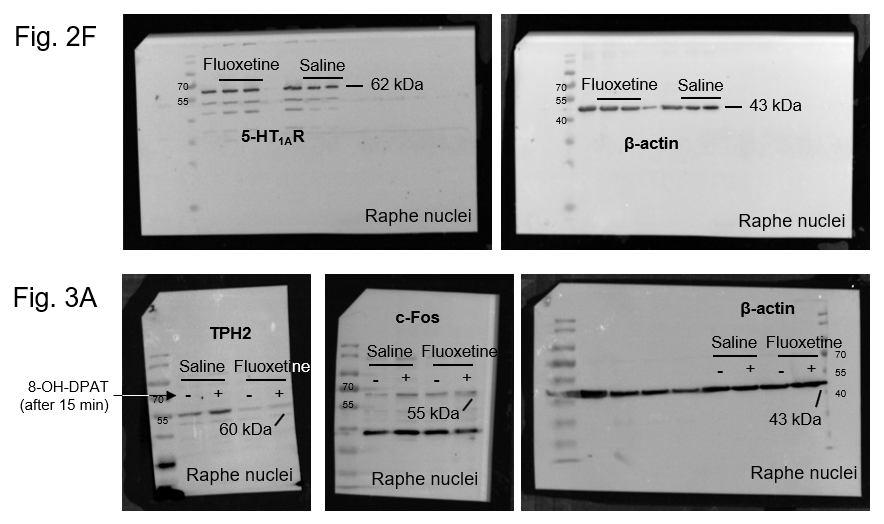


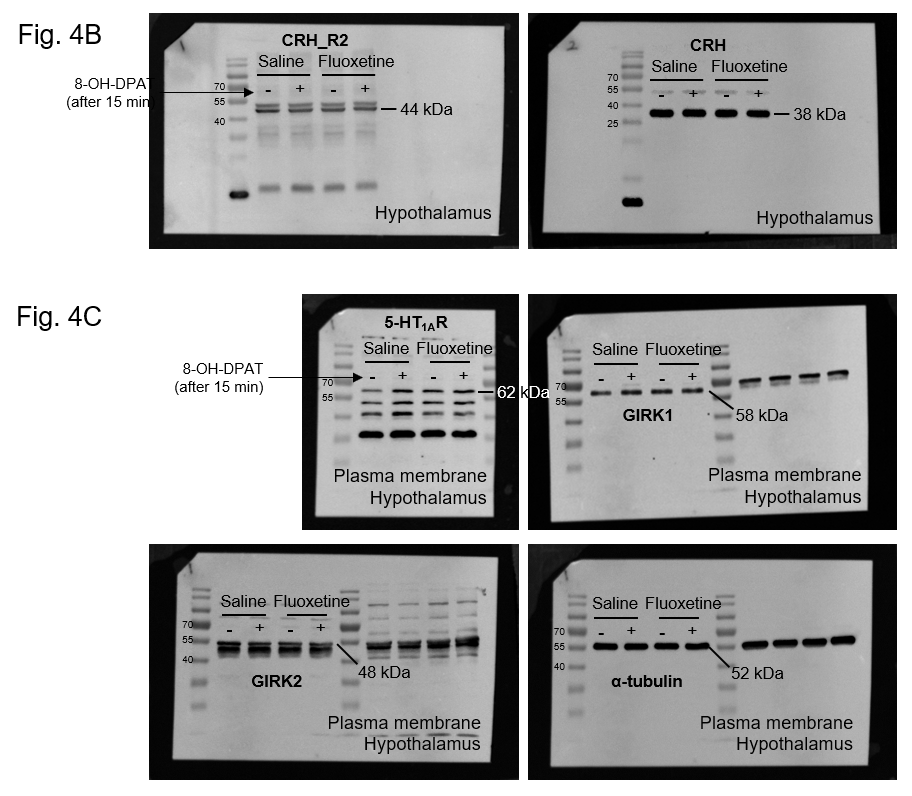


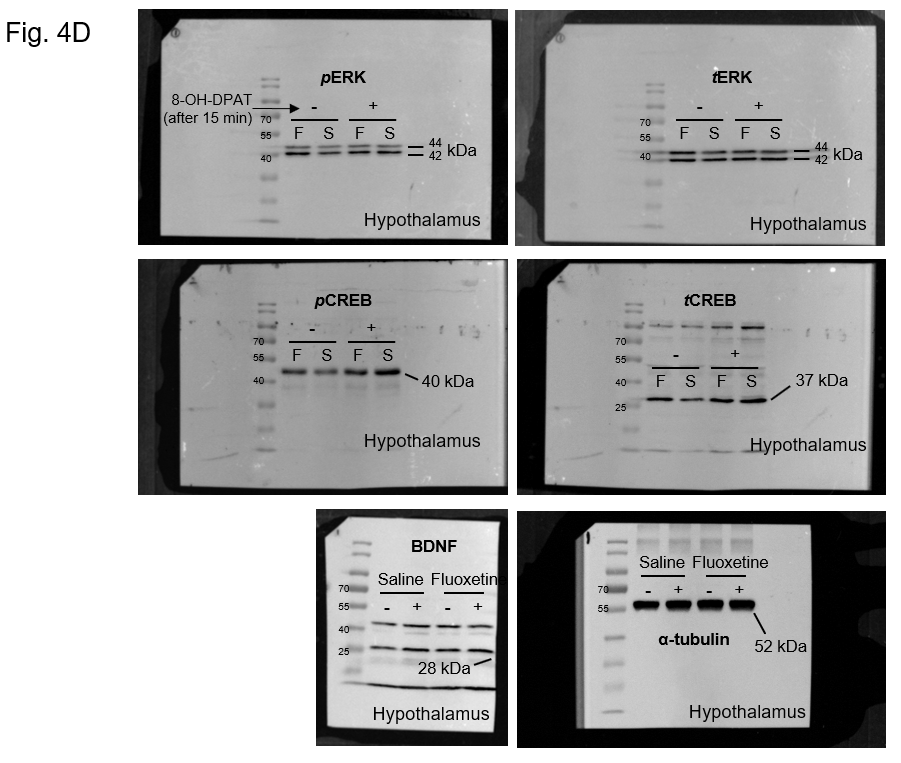


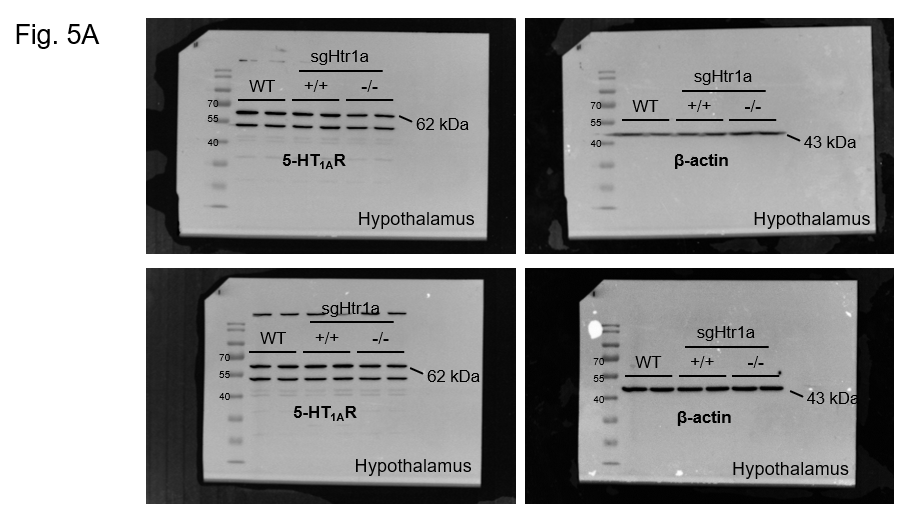


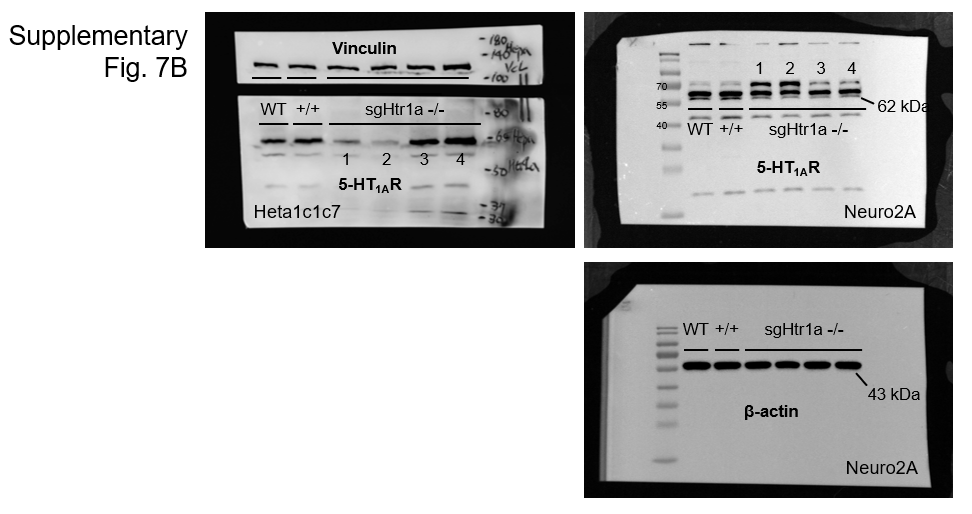

Supplement: Supplementary file 1 — Additional file 1. Supplementary Materials. Fig. S1. Experimental designs. Fig. S2. Fatigue and ME/CFS-like behaviors in female mice. Fig. S3. Quantifications of hypothalamic protein expression. Fig. S4. Microglial and astrocytic activity and plasma TGF-β1 levels. Fig. S5. Body weight, food intake, and the levels of liver enzymes. Fig. S6. Cognition and hippocampal neurogenesis. Fig. S7. CRISPR/Cas9-mediated Htr1a knockdown. Table S1. Target sequence for Mus musculus Htr1a. Table S2. Sequence of the primers used in real-time PCR analysis. [file 12967_2023_4808_MOESM1_ESM.docx]
